# Activation Energy of Extracellular Enzymes in Soils from Different Biomes

**DOI:** 10.1371/journal.pone.0059943

**Published:** 2013-03-25

**Authors:** J. Megan Steinweg, Sindhu Jagadamma, Joshua Frerichs, Melanie A. Mayes

**Affiliations:** 1 Biosciences Division, Oak Ridge National Laboratory, Oak Ridge, Tennessee, United States of America; 2 Environmental Sciences Division, Oak Ridge National Laboratory, Oak Ridge, Tennessee, United States of America; 3 Climate Change Science Institute, Oak Ridge National Laboratory, Oak Ridge, Tennessee, United States of America; 4 Civil and Environmental Engineering, University of Tennessee, Knoxville, Tennessee, United States of America; Missouri University of Science and Technology, United States of America

## Abstract

Enzyme dynamics are being incorporated into soil carbon cycling models and accurate representation of enzyme kinetics is an important step in predicting belowground nutrient dynamics. A scarce number of studies have measured activation energy (E_a_) in soils and fewer studies have measured E_a_ in arctic and tropical soils, or in subsurface soils. We determined the E_a_ for four typical lignocellulose degrading enzymes in the A and B horizons of seven soils covering six different soil orders. We also elucidated which soil properties predicted any measurable differences in E_a_. β-glucosidase, cellobiohydrolase, phenol oxidase and peroxidase activities were measured at five temperatures, 4, 21, 30, 40, and 60°C. E_a_ was calculated using the Arrhenius equation. β-glucosidase and cellobiohydrolase E_a_ values for both A and B horizons in this study were similar to previously reported values, however we could not make a direct comparison for B horizon soils because of the lack of data. There was no consistent relationship between hydrolase enzyme E_a_ and the environmental variables we measured. Phenol oxidase was the only enzyme that had a consistent positive relationship between E_a_ and pH in both horizons. The E_a_ in the arctic and subarctic zones for peroxidase was lower than the hydrolases and phenol oxidase values, indicating peroxidase may be a rate limited enzyme in environments under warming conditions. By including these six soil types we have increased the number of soil oxidative enzyme E_a_ values reported in the literature by 50%. This study is a step towards better quantifying enzyme kinetics in different climate zones.

## Introduction

In recent years, increasingly complex and realistic soil carbon models explicitly include microbial processes [1]–[5]. However, most soil carbon models remain largely simplified constructs because of the difficulty in ascertaining microbial responses to different variables [6]. One difficulty in modeling the soil carbon system is understanding the microbial mechanisms that are influenced by temperature, such as enzymatic reactions (extra- and intracellular), diffusion of substrates, and microbial substrate utilization efficiency [7]–[9]. The temperature response of reactions at all scales of life can be determined by calculating the activation energy (E_a_) [10]–[15]. Activation energy is the difference in energy between reactants and the transitional species that decay into products, and this determines the change in a reaction's rate with temperature. However, estimating this parameter for enzymatic reactions is challenging because activity and E_a_ are specific to the type of enzyme [16], *in situ* temperature range, substrate, and other edaphic characteristics under consideration [9].

At the ecosystem scale, extracellular enzyme activity is influenced by organic matter abundance and composition [17]. Lignocellulose, a main component of plant litter is comprised primarily of cellulose, hemicellulose, and lignin [14] and is broken down extracellularly by a suite of enzymes produced by many organisms [9]. In order to convert cellulose into glucose, three categories of hydrolases are produced by microbes: cellobiohydrolase, endoglucanase and β-glucosidase [18]–[19]. Lignin is broken down by a class of oxidoreductases called ligninases [20]. Typical ligninases include peroxidases and phenol oxidases [17], [21]. Enzymes that depolymerize high molecular weight compounds, such as lignin require more enzymatic steps and have been shown to have higher E_a_ than enzymes that break down simpler compounds [16], [22]. A higher E_a_ for complex compounds indicates that there may be a disproportionate effect of increasing temperatures on the depolymerization of high molecular weight components of organic matter [6].

*In situ* temperature also influences the temperature optima of enzymes. Several studies have demonstrated that enzymes produced by microorganisms in colder climates have lower temperature optima [23]–[24]. The stability of an enzyme's structure is dependent on the *in situ* temperature range, with enzymes in colder climates having more flexible structures than enzymes in warmer climates [25]–[26]. The ability of enzymes to change structural conformation with temperature [27], thus altering the active site, could impact the E_a_ of enzymatic reactions in soils from different climates because isoenzymes for the same reaction do have different substrate affinities [28] and may have different E_a_. Extracellular enzymes can be sorbed to clays [29]–[30] which can affect their activity. Studies have shown both increases and decreases in activity once enzymes become immobilized on clays [31]–[32]. If sorption alters the structure of the enzymes, particularly the active site, it could change the kinetic properties of the enzyme [33].

Surprisingly, a scarce amount of studies have been carried out to determine the E_a_ of soil enzymes [16], [34]–[35], especially for oxidative enzymes. More often, it is easier to find studies carried out on pure enzymes, however these studies do not indicate how edaphic conditions might alter E_a_ [36]–[43]. Because of the paucity of data we chose to determine the E_a_ for four typical lignocellulose degrading enzymes in surface and subsurface horizons of seven soils covering six different soil orders. We also elucidated which soil properties predicted any measurable differences in E_a_. We hypothesized that E_a_ for all enzymes would be greatest in cold regions (arctic and subarctic), followed by temperate and tropical regions. Tropical regions have very stable temperatures so that enzymes may be adapted to the constant warm temperatures whereas temperatures fluctuate daily and seasonally in the temperate and cold regions. In addition, we hypothesized that (2) the ambient temperature 30 days prior to sampling would influence the enzyme pool, so that potential enzyme activity would be lower in soils collected during warmer periods compared to colder periods due to enzymatic efficiency, and (3) E_a_ for oxidative enzymes would be greater than hydrolytic enzymes, regardless of climate regions, due to the difference in substrate complexity.

## Materials and Methods

### Site Descriptions

Soil samples were collected from a broad range of climatic zones across the western hemisphere (Table 1). From each location, two or three soil samples were taken from both A and B horizons, composited and 2 mm sieved. From the seven soils tested, six soil orders (Alfisol, Andisol, Gelisol, Mollisol, Oxisol, and Utlisol) and four major climate zones (arctic, subarctic, temperate, and tropical) were represented. No specific permits were required to collect soil samples from the field locations, which were on public, non protected land. In the case of the Icelandic site The Environment Agency of Iceland was informed of the soil sampling and according to regulation B, no. 520/1975 a permit is not required for soil sampling for scientific purposes. All necessary shipping permits were obtained for the described field samples, a USDA APHIS quarantine permit for shipment of soils from outside the United States and a compliance agreement for the shipment of domestic soils. The temperate Ultisol and temperate Mollisol are both US-DOE sites, and the arctic Gellisol is a US-ACE site. The sample collection did not involve or harm any endangered or protected species.

**Table 1:** Soil characteristics and environmental variables.

Soils	Location	Order	Sample Year	Horizon	depth (cm)	Clay (%)	pH	Total C (%)	avg T (°C)	GWC	MAT (°C)	MAP (mm)
Arctic 1	Fairbanks, AK	Gelisol	Dec-10	Active	0–30	13	7.03	2.54	−12.0	0.362	−2.9	572
				Permafrost	50–75	13	8.05	1.82		0.453	−2.9	572
Subarctic 1	Krýsuvík, Iceland	Andisol	July-11	A	0–15	12	5.84	8.49	8.6	0.775	5.0	1600
				B	35–55	10	6.13	8.14		1.053	5.0	1600
Temperate 1	Kane, IL	Mollisol	July-11	A	0–15	35	6.70	3.72	19.9	0.354	11.3	996
				B	55–70	26	6.80	1.66		0.291	11.3	996
Temperate 2	Gibson, TN	Alfisol	April-11	A	0–15	29	5.50	1.06	12.2	0.217	16.9	1381
				B	50–75	32	5.80	0.52		0.12	16.9	1381
Temperate 3	Blount, TN	Ultisol	May-11	A	0–10	25	6.17	1.97	16.6	0.239	14.7	1225
				B	40–60	45	5.11	0.26		0.203	14.7	1225
Tropical 1	Lavras, Brazil	Oxisol	March-11	A	0–12	67	4.42	5.85	24.0	0.299	19.3	1343.3
				B	42–65	79	4.68	2.33		0.272	19.3	1343.3
Tropical 2	Lavras, Brazil	Ultisol	March-11	A	0–10	45	5.42	3.17	24.0	0.24	19.3	1343.3
				B	37–50	42	5.17	1.07		0.227	19.3	1343.3

*Total C = total carbon, avg T = average air temperature (°C) for the month preceding sampling, GWC = gravimetric water content, MAT = mean annual temperature, MAP = mean annual precipitation.*

### Soil Processing

Field moist soil samples were composited, 2 mm sieved, and then stored at −10°C for a maximum of 1 week until enzyme analyses could be performed. Gravimetric water content (GWC) was determined in triplicate by oven drying the pre-weighed subsamples for 24 hr at 105°C, and then reweighing each subsample. Total carbon and nitrogen analyses were performed on air dried, ground soil using a LECO TruSpec CN analyzer (LECO Corp., St. Joseph, MI). Particle size analysis was performed using the Buoycous hydrometer method [44]. Soil pH was measured on the supernatant of a 5 mM CaCl2 solution in a 2∶1 solution to solid ratio.

### Enzyme Assays

Enzymatic assays were performed, in 96-well microplates on two hydrolases, β-glucosidase (BG, EC 3.2.1.21) and cellobiohydrolase (CB, EC 3.2.1.91), and two oxidases, peroxidase (PER, EC 1.11.1.7) and phenol oxidase (POX, EC 1.10.3.2) [45]. Each assay was performed at 4, 21, 30, 40, and 60°C in 50 mM pH 5 sodium acetate buffer. Typically assays are performed at the *in situ* soil pH, however Wang et al. (2012b) have demonstrated that most soil enzymes have pH optima around 5. Also, oxidative enzyme activity is very difficult to measure at a higher pH due to abiotic oxidation of substrates, which can lead to incorrect calculation of E_a_. Through laboratory trials, it was determined that a pH 5 buffer would be an adequate pH for these soils despite the wide range of *in situ* soil pH (Table 1). For every soil, three 1 g subsamples were taken to represent the heterogeneity at the site. Each subsample was mixed with 125 mL of 50 mM pH 5 acetate buffer were mixed with a hand blender for two minutes. The suspension was then added to a 150 mm petri dish and maintained using a magnetic stir rod.

The hydrolase assays were performed in replicates of eight in black 96-well microplates. The blank wells on each plate received 250 µL of acetate buffer, reference-standard wells received 200 µL of acetate buffer and 50 µL of 10 µM 4-methylumbelliferone (MUB) standard, and negative-control wells received 200 µL acetate buffer and 50 µL 200 µM 4-MUB-linked substrates (4-MUB-β-D-glucoside and 4-MUB-β-D-cellobioside). For each soil, quench-control wells received 200 µL of soil suspension and 50 µL 10 µM 4-MUB standard, sample control wells received 200 µL soil suspension and 50 µL of 50 mM acetate buffer. Activity assay wells received 200 µL of soil suspension and 50 µL of 200 µM 4-MUB-linked substrate. After incubating for 2 h, each black microplate received 10 µL 0.5N NaOH in every well in order to raise the pH and enhance the fluorescence to a detectable level. Fluorescence was then detected at an excitation wavelength 365 nm, emission wavelength 450 nm, and sensitivity of 50 with a BioTec Synergy™MX Multi-Mode Microplate Reader.

The oxidase assays were performed in clear 96-well UV microplates. For each plate, 8 blank wells received 250 µL of acetate buffer and 16 reference-standard wells received 200 µL acetate buffer and 50 µL of L-3,4-dihydroxyphenylalanine (L-DOPA). For each soil, 8 homogenate control wells received 200 µL of soil suspension and 50 µL of acetate buffer and 16 assay wells received 200 µL soil suspension and 50 µL L-DOPA. Additionally, each well of the peroxidase assay plates received 10 µL of 0.3% H_2_O_2_. After incubation for 24 h, clear microplates were read spectrophotometrically at 460 nm with a BioTec Synergy™MX Multi-Mode Microplate Reader.

### Data Analysis

For enzymatic assays, the activities for both hydrolases and oxidases were expressed as nmol of substrate converted per g dry soil per h (nmol g dry soil^−1^ h^−1^). An Arrhenius plot was created to estimate activation energy according to the Arrhenius equation:
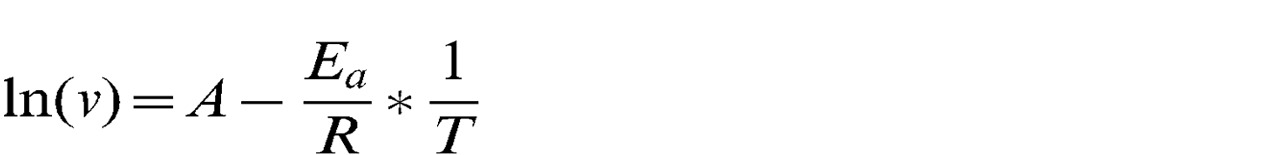
(1)where A, a constant, is the frequency factor, E_a_ is the activation energy, R is the gas constant and T is the absolute temperature (°K)

Relationships between E_a_ and environmental parameters in Table 1 were determined using both linear and polynomial regressions. In addition, PROC GLM (SAS Inc., Cary NC) was used to determine if biome type had a significant effect on E_a_(*P*<0.05), using the three enzyme subsamples from each location.

## Results

In most cases, β-glucosidase E_a_ tended to be higher in the B horizon compared to the A horizon horizon, except in the arctic system where they were similar (Table 2; *P*<0.05). Phenol oxidase E_a_, was consistently greater in the A horizon than B horizon soil in the tropics (*P*<0.05). Cellobiohydrolase and peroxidase E_a_ showed no discernible trend with soil depth.

**Table 2:** Activation Energies (kJ mol^−1^) with standard error in parentheses (analytical replicates n = 8 for BG, CB; n = 16 for PER, POX) for extracellular enzymes. “*” indicated n = 1.

Soil		E_a_ (kJ/mol)							
	Horizon	BG		CB		PER		POX	
Arctic	A	35.4	(1.34)	39.4	(3.93)	12.7	(0.52)	81.8	(7.38)
	P	34.8	(1.05)	18.7	(4.22)	21.3	(0.92)	74.2	(3.16)
Subarctic	A	36.5	(0.73)	38.6	(0.59)	21.2	(2.16)	45.7	(5.56)
	B	52.2	(2.08)	41.5	(0.62)	22.4	(2.20)	39.4	(10.10)
Temperate 1	A	40.9	(1.46)	38.0	(1.05)	64.9	(2.24)	102.0	(9.22)
	B	49.4	(2.48)	21.2	(9.97)	28.0	(6.50)	94.8	(6.81)
Temperate 2	A	31.0	(0.69)	43.4	(0.81)	25.4	(1.80)	49.5	(5.88)
	B	40.9	(2.48)	39.9	(7.48)	19.8	(1.82)	47.5	(3.75)
Temperate 3	A	51.5	(2.17)	53.6	(2.51)	28.8	(1.69)	73.2	(6.09)
	B	58.8	(4.98)	46.7	(2.36)	54.2	(8.84)	29.0	(4.58)
Tropical 1	A	47.8	(0.93)	50.5	(7.58)	26.5	(4.25)	47.7	(10.00)
	B	56.6	(2.66)	47.0	*	47.1	(4.58)	27.1	(11.90)
Tropical 2	A	39.3	(1.51)	42.5	(2.27)	58.3	(5.12)	82.5	(9.96)
	B	42.8	(1.91)	43.3	(2.46)	22.8	(3.09)	45.5	(3.55)

**Figure 1:**
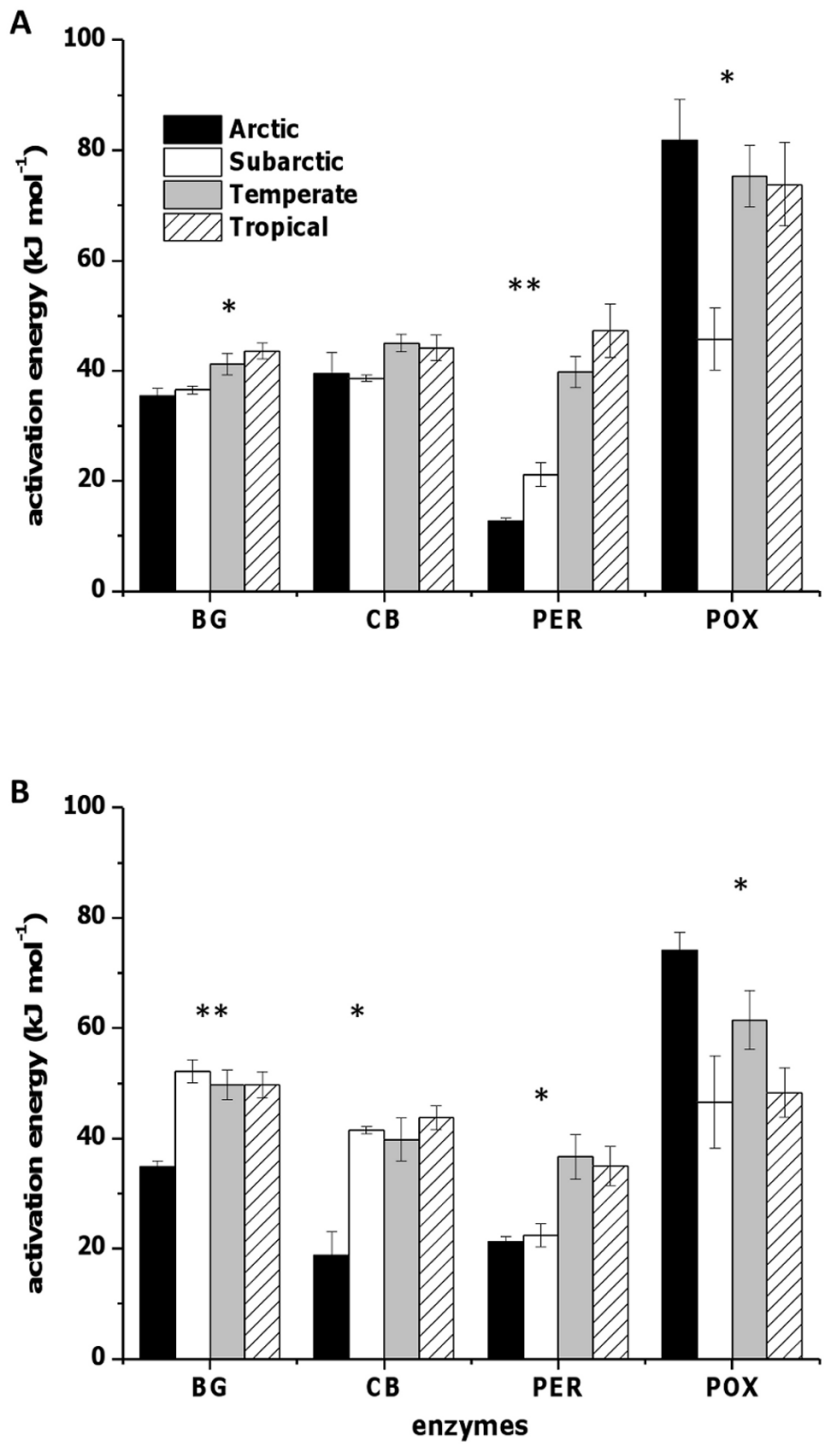
Activation energy for hydrolytic and oxidative enzymes. E_a_ in (a) A horizon and (b) B horizon for β-glucosidase (BG), cellobiohydrolase (CB), peroxidase (PER) and phenol oxidase (POX). Activation energy calculated from three subsamples taken from each soil and depth combination. The number of study locations per biome were tropical n = 2; temperature n = 3; subarctic n = 1; arctic n = 1. The “*” indicates a significant effect of biome with a 0.05≤*P*≤0.1 and “**” indicates a significant effect of biome with a *P*≤0.01.

E_a_ for all enzymes was affected by biome type (Figure 1; *P*<0.1 and 0.05). In the A horizon, E_a_ for the hydrolases was similar for arctic and subarctic biomes, while in the B horizon the hydrolase's E_a_ for the subarctic was more similar to temperate and tropical biomes. In the A horizon, within a biome the E_a_ of the hydrolytic enzymes was similar, whereas the E_a_ of the two oxidative enzymes were very different from each other. In the B horizon, the E_a_ of both hydrolases (β-glucosidase and cellobiohydrolase) were always lower in the arctic biome (Alaska) than subarctic, temperate and tropical biomes (*P*<0.1 and 0.05). Phenol oxidase E_a_ was similar in arctic and temperate biomes in both horizons, and lowest in the subarctic biome (*P*<0.1).

The E_a_ of β-glucosidase and cellobiohydrolase have positive relationships with average air temperature in the month preceding sampling and mean annual temperature (Table 3). Soil pH had a strong negative relationship on the E_a_ of hydrolases in the B horizon, and a strong positive relationship with phenol oxidase E_a_ in both horizons. The relationship between clay and E_a_ was positive for β-glucosidase and peroxidase in both horizons, but negative for phenol oxidase E_a_ in the B horizon. Significant regressions are shown in Supplemental Figure 1.

**Table 3:** Linear and polynomial regression statistics relating E_a_ for four enzymes to four different environmental variables.

		A HORIZON				B HORIZON			
enzyme	variable	regression	r^2^	*F*	*P*	regression	r^2^	*F*	*P*
β-glucosidase	clay	y = 0.21x+31.21	0.28	3.35	0.13	y = 0.25x+35.76	0.22	2.67	0.16
	pH					y = −4.87x+75.01	0.54	8.09	0.04
	avg T	y = 31.8−0.04x+0.02x^2^	0.35	2.62	0.19	y = 0.40x+40.44	0.54	8.23	0.04
	MAT					y = 41.54+1.96x−0.09x^2^	0.56	4.87	0.08
cellobiohydrolase	clay								
	pH					y = −4.95x+71.1	0.72	16.43	0.01
	avg T					y = 34.74+1.03x−0.03x^2^	0.71	7.17	0.07
	MAT	y = 0.39x+36.50	0.37	4.49	0.09	y = 30.57+2.58x−0.09x^2^	0.71	8.30	0.04
peroxidase	clay	y = 1.01x+0.99	0.46	6.15	0.06	y = 24.44−0.34x+0.008x^2^	0.48	3.82	0.12
	pH								
	avg T	y = 0.83x+22.28	0.62	11.13	0.02				
	MAT	y = 1.2x+16.56	0.44	5.73	0.06				
phenol oxidase	clay					y = −0.79x+79.75	0.31	3.73	0.11
	pH	y = 14.71x−21.78	0.23	2.77	0.16	y = 12.45x−2.41	0.61	10.40	0.02
	avg T								
	MAT					y = −1.37x+69.98	0.34	4.15	0.10

*Data are shown for A and B horizon regressions in Supplemental* *Figure 1* *, avg T = average air temperature (°C) for the month preceding sampling; MAT = mean annual temperature (°C).*

**Figure 2:**
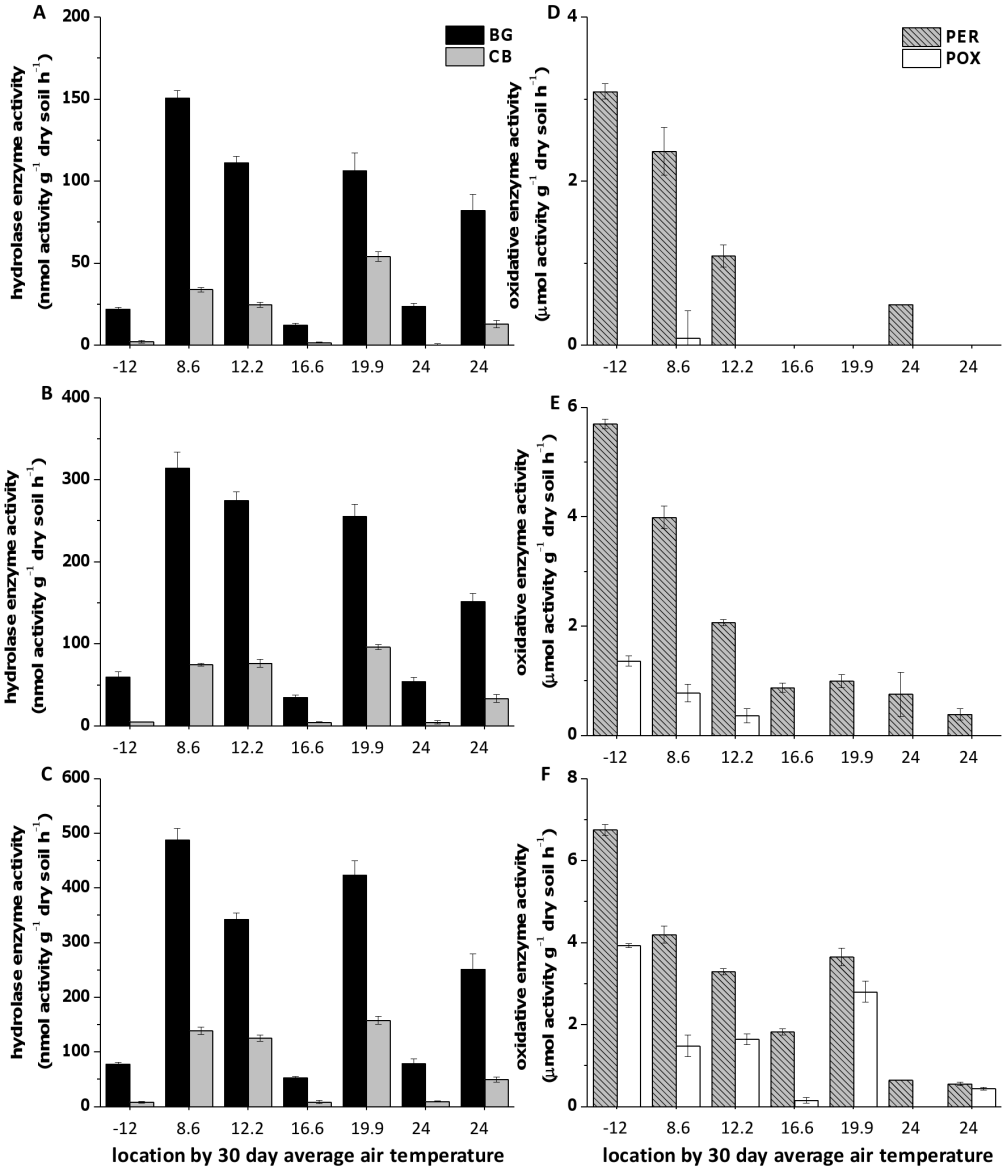
Hydrolytic and oxidative enzyme activities in the A horizon. Enzyme activities at (a) 4°C, (b) 21°C, and (c) 30°C for hydrolytic enzyme activities in order by average air temperature 30 days prior to sampling. Oxidative enzyme activity at (d) 4°C, (e) 21°C, and (f) 30°C in order by average air temperature 30 days prior to sampling.

There was no clear relationship between potential hydrolase activities and average air temperature in the month preceding sampling (Figure 2, A horizon only). The arctic location, one temperate, and one tropical location all showed low potential β-glucosidase and cellobiohydolase. Potential activity at 4, 21, 30, 40 and 60°C were used to calculate E_a_ but activity for 40 and 60°C are not shown in Figure 2 because those temperatures are well outside the range of native soil temperatures in all locations and in some locations no activity was detected despite being present at lower temperature assays, likely due to enzyme inactivation at high temperatures. Activity increased for β-glucosidase (42–68%), cellobiohydrolase (37–57%), peroxidase (0–75%), and phenol oxidase (0–55%) in the A horizon for assays performed between 4°C and 21°C. β-glucosidase activity was always the highest followed by cellobiohydrolase activity, peroxidase activity and phenol oxidase activity. Phenol oxidase activity was not detected at 4° and 21°C in the temperate and tropical locations but was detected in the arctic and subarctic locations. Enzyme activity for both oxidative enzymes showed a distinct trend with decreased activity as average air temperature in the month preceding sampling increased (Figure 2; *P*<0.2). B horizon enzyme activity increased with assay temperature, however there was no clear trend for activity with average air temperature (data not shown).

## Discussion

This study is one of the few that has looked at the E_a_ for hydrolytic and oxidative enzymes across soil orders and soil depth. In particular, by including arctic and tropical soils we have increased the breadth of E_a_ values beyond the temperate zone. Despite the importance of arctic and tropical ecosystem to the carbon cycle, there is little information regarding E_a_ of hydrolase and oxidative enzymes [46]–[47]. Overall, the E_a_ values for the hydrolases in all climatic zones were similar or slightly higher than other reported values [16], [46], [48], with average E_a_ values of 44 and 40.3 for β-glucosidase and cellobiohydrolase across all biomes, respectively. The cellobiohydrolase reaction occurs before the β-glucosidase reaction, but they had similar E_a_ indicating no differential response to temperature or a rate limiting step.

In several cases the E_a_ in the B horizon was higher than other reported values from A horizon soils, but B horizon soils are often not considered in enzyme studies, so we have little data for direct comparison. Most enzyme studies consider only the portion of soil near the surface (top 15 cm, usually A horizon), but there is a large portion of the terrestrial carbon stored below. It is unclear why enzyme studies tend to ignore enzymes dynamics below the first 15–20 cm, perhaps it is assumed that enzymes in the B horizon would respond similarly to those in the A horizon or that they are less affected by environmental disturbance due to their depth.

Across enzymes there was no consistent relationship between E_a_ and the environmental variables we measured. We found that in the A horizon there were fewer relationships between E_a_ and the environmental variables we used (MAT, pH, clay and average 30 day temperature) compared to the B horizon. Only the positive linear relationship between phenol oxidase E_a_ and pH was maintained in both A and B horizons. There are other environmental variables (mineral content, clay type) not measured in this study that could be measured in the future to determine their influence if any over E_a_.

The information to date on E_a_ for oxidative enzymes is scant from soil environments [14], [47]–[48] with most of E_a_ estimates coming from pure cultures of isolated enzymes [41]. A total of 11 values for phenol oxidase E_a_ and 14 values for peroxidase E_a_ were identified in [46], with most of those values coming from one study [48]. Previously estimated values for peroxidase E_a_ in soil samples ranged from 30.5 kJ mol^−1^ [48] up to 60 kJ mol^−1^ [14] with an average of 54 kJ mol^−1^ [47]. The value for peroxidase E_a_ in this study averaged across all climate regions was lower, 32 kJ mol^−1^, than previously estimated, but particularly in the arctic and subarctic regions, which had an average E_a_ of 19 kJ mol^−1^. Phenol oxidase activity ranged from 29–102 kJ mol^−1^ at our study sites and averaged 59.5 KJ mol^−1^ which was similar to previously recorded phenol oxidase E_a_ values 37–57 kJ mol^−1^ [48], with an average of 54 kJ mol^−1^ [47]. The low peroxidase E_a_ in colder regions was counter to our hypothesis that enzymes in colder regions would be more temperature sensitive than those in warmer region, however this does not hold true for phenol oxidase. The large difference in E_a_ between peroxidase and phenol oxidase indicate that not all oxidative enzymes respond similarly to temperature, whereas the two hydrolytic enzymes had similar temperature responses. Grouping oxidative enzymes together, as we did in our hypothesis, was incorrect because peroxidases appear to be much less temperature sensitive than hydrolytic enzymes, whereas phenol oxidases are much more temperature sensitive.

In the soils we used, phenol oxidase had a greater E_a_ than peroxidase indicating that the phenol oxidase reactions are more sensitive to temperature increases than peroxidase reactions, thus phenol oxidase reaction rates may increase more than peroxidase reaction rates with warming. Peroxidase also had very low E_a_ compared to the hydrolytic enzymes, indicating that the reaction rate is less sensitive to changes in temperature. In this study, peroxidase was the dominant oxidative enzyme in most environments, thus if temperatures rise, it may be the rate limiting step in decomposition because of its reduced sensitivity to temperature.

Enzyme activity for both oxidative enzymes showed a distinct trend with decreasing activity as the 30 day average temperature increased in accordance with our hypothesis that there would be lower potential enzyme activity in soils collected during warmer time periods because of increased enzymatic efficiency (Figure 2d–f; *P<0.2*). There was very little measurable peroxidase and no phenol oxidase activity in the soils from the warmer locations during the 4°C incubations and no measurable phenol oxidase activity in soils warmer locations during the 21°C incubations. This may be due to the structural conformation of the peroxidase and phenol oxidase enzymes. Enzymes in warmer locations tend to have more rigid or stable conformations whereas enzymes in colder environments tend to have more fluid conformations [25], [26]. The initially rigid conformation of the oxidative enzymes from warmer climates may have made it more difficult for enzymes to interact with substrates in the colder incubation temperature because the low temperature increased the rigidity of the enzyme. However, we do not see this clear trend for hydrolytic enzyme activity.

Enzyme activity and thus E_a_ calculations are made by adding simple substrates to soil slurries. The substrates are similar in structure to the substrates depolymerized in nature by enzymes but different in complexity. As mentioned earlier, plant material is made up of many different substrates but primarily lignin and cellulose which form a lignocelluloses complex. The lignocellulose complex has the lignin and cellulose intertwined so that enzymes need to work in conjunction to break down the material. The substrates we add in assays are single substrates, not in a complex, so the enzymes likely break down the substrates at a faster rate than in nature. Since E_a_ is calculated using activity rates the use of single substrates may decrease the E_a_ over what occurs in nature when a consortium of enzymes are required to complete the depolymerization of lignocelluloses complexes.

This study is by no means a comprehensive list of enzyme E_a_ across the globe, out of the thirty possible enzymes to be assayed we selected only four, but we did select enzymes representative of two major groups, hydrolases and oxidases. Enzyme activity has been measured in arctic and tropical biomes, however the E_a_ for enzymes in these systems has rarely been measured before [46], [49]. By including these six soil types we have increased the number of soil oxidative enzyme E_a_ values reported in the literature by fifty percent. This study is a step towards better understanding and comparing enzyme kinetics in different climate zones. Also, it points out that the classification of enzymes by reaction types may not be indicative of their responses to temperature. Enzyme dynamics are being incorporated into models [5] and having accurate representation of enzyme kinetics from different regions is an important step in predicting nutrient dynamics.

## Supporting Information

Figure S1 **Activation energy for all enzymes in both soil horizons.** E_a_ in the A horizon (a–d), B horizon (e–h), and combined A and B horizons (i–l) across four soil characteristics: clay, pH, average air temperature (°C) for the month preceding sampling, and mean annual temperature (MAT). Significant and marginally significant linear and polynomial regressions are shown for each enzyme and each soil characteristic, *P*<0.2.
(TIF)
