# Supplementary figures and images for: Activation Energy of Extracellular Enzymes in Soils from Different Biomes
Source: PLoS One. 2013 Mar 25;8(3):e59943. doi: 10.1371/journal.pone.0059943 (PMC3607567; doi:10.1371/journal.pone.0059943)

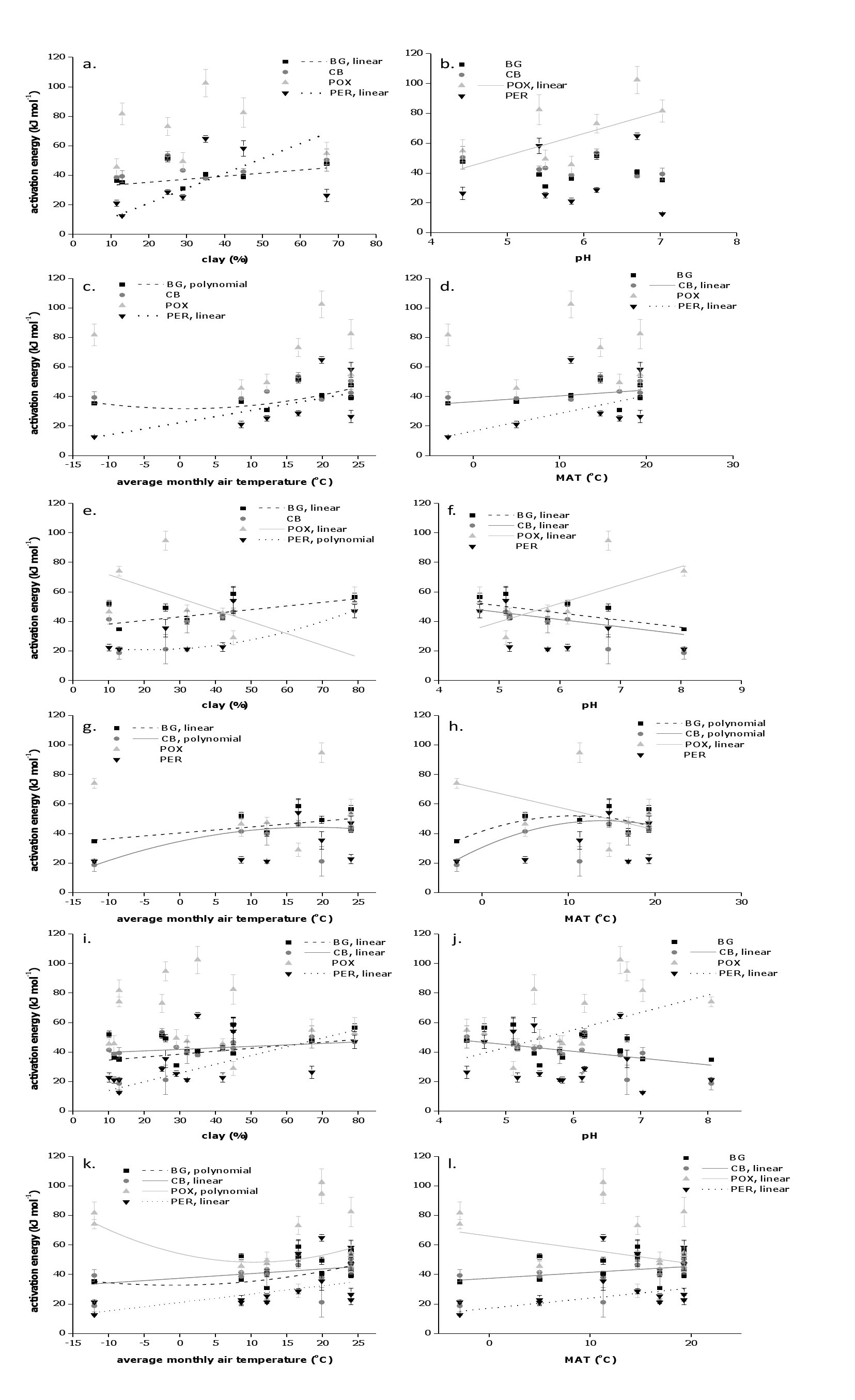

Supplement: Figure S1 — Activation energy for all enzymes in both soil horizons. Ea in the A horizon (a–d), B horizon (e–h), and combined A and B horizons (i–l) across four soil characteristics: clay, pH, average air temperature (°C) for the month preceding sampling, and mean annual temperature (MAT). Significant and marginally significant linear and polynomial regressions are shown for each enzyme and each soil characteristic, P<0.2. (TIF) [file pone.0059943.s001.tif]
